# The Aftermath of Bariatric Surgery: Can the Average Emergency Surgeon Deal with Its Complications? Comment on Zawadzka et al. Current Knowledge and Perceptions of Bariatric Surgery among Diabetologists and Internists in Poland. *J. Clin. Med.* 2022, *11*, 2028

**DOI:** 10.3390/jcm11123401

**Published:** 2022-06-14

**Authors:** Chrysanthi Papageorgopoulou, Konstantinos Nikolakopoulos, Charalampos Seretis

**Affiliations:** 1 Surgical Directorate, General University Hospital of Patras, 265 04 Rio, Greece; chrisanthi.papageorg@gmail.com (C.P.); konstantinosn@yahoo.com (K.N.); 2 Surgical Directorate, George Eliot Hospital NHS Trust, Warwickshire CV10 7DJ, UK

We read with great interest the article by Zawadzka et al. [1], which provided a very interesting overview regarding the knowledge of a surveyed group of internal medicine physicians and diabetologists with respect to the indications, exclusion criteria and perioperative management of bariatric surgery patients. Putting the survey results in a nutshell, the authors concluded that although these healthcare providers had a general understanding of these issues, there was a lack of detailed knowledge which would enable a fast-track referral and smooth medical follow-up system. The latter was highlighted in the expressed wish for structured relevant training and the establishment of communicating pathways with their tertiary bariatric surgery units, a fact which is emerging as a universal need [2]. Bearing the abovementioned issues in mind, and addressing these findings from a surgical perspective, we would like to stress the issue of an urgent need for the similar education of general surgeons regarding bariatric surgery, focusing on the management of its complications.

With the global increase in the acceptance of bariatric or metabolic surgery as a potentially curative procedure for morbid obesity and its life-threatening consequences, a new field of digestive surgery has been created. Apart from the purely technical aspect of the surgical procedures, digestive surgeons need to have a broad understanding of the pathophysiological effects of these operations, as well as a high level of awareness when it comes to the management of the early and late complications that can follow these procedures. Due to the creation of a new digestive tract anatomy, specialist training is required to allow the accurate interpretation of paraclinical investigations, such as computed tomography scans, as well as to enable the formulation of accurate operative or non-operative treatment plans, all in a timely fashion. The gap in non-bariatric specialist surgeons’ knowledge and clinical confidence regarding the abovementioned issues was clearly highlighted in a recent large-scale survey among members of the World Society of Emergency Surgery [3]. Summarizing some of the key findings of this survey, almost half of the participating emergency surgeons reported that they could not confidently identify the type/site of the complication that occurred, even after the completion of abdominal diagnostic scans. Of note, the surveyed emergency surgeons reported that almost 50% of these re-operated patients required admission to the intensive care unit, a fact which is indicative of the impact of the physiological harm they had sustained. Therefore, it is evident that it is high time for the provision of structured educational programmes not only to physicians who deal with bariatric surgery patients, but also for general surgeons who have not received specialist training in bariatric surgery in the context of their residency and/or fellowships. Above all, as is the case, for instance, with complex hepatobiliary or advanced surgical oncology cases, the management of acutely unwell post-bariatric surgery patients should be reserved for specialists in the field at the tertiary level.
